# Supplementary material for: Ethanolaminephosphate cytidyltransferase is essential for survival, lipid homeostasis and stress tolerance in Leishmania major
Source: bioRxiv. 2023 Jan 11:2023.01.10.523530. Preprint. [Version 1] doi: 10.1101/2023.01.10.523530 (PMC9882048; doi:10.1101/2023.01.10.523530)
Supplement: 1 [file NIHPP2023.01.10.523530v1-supplement-1.pdf]

bioRxiv preprint doi: https://doi.org/10.1101/2023.01.10.523530; this version posted January 11, 2023. The copyright holder for this preprint (which was not certified by peer review) is the author/funder, who has granted bioRxiv a license to display the preprint in perpetuity. It is made available under aCC-BY 4.0 International license.

SUPPORTING INFORMATION

Figure S1. Figure S1. Alignment of EPCT amino acid sequences from *Saccharomyces cerevisiae* (*Sc*, Genbank: P33412), *Plasmodium falciparum* (*Pf*, Plasmodb: PfDd2_130053500), *Leishmania major* (*Lm*, Tritrypdb: LmjF.32.0890), *Arabidopsis thaliana* (*At*, TAIR: AT2G38670.1), and *Homo sapiens* (*Hs*, Genbank: Q99447) using Clustal Omega. Asterisks (*): fully conserved residues; colons (:) highly similar residues; periods (.): moderately similar residues. Color code for amino acids: red-nonpolar; green-polar; blue-acidic; purple-basic.

Figure S2. Detection of EPCT activity with thin layer chromatography (TLC). (**A**) EtN-P or CDP-EtN (40 nmol each) are resolved by TLC as described in *Materials and Methods*. The plate was dried and sprayed with 0.2% ninhydrin to show the positions of EtN-P and CDP-EtN. (**B**) Mouse liver lysates (boiled and un-boiled, two repeats each) were incubated with [^{14}C]-EtN-P at room temperature, followed by TLC analysis and signals were detected by autoradiography.

Figure S3. Chromosomal *EPCT*-null mutants cannot be generated without a complementing episome. Genomic DNA samples from *L. major* FV1 WT, LV39 WT, *EPCT+/-* (#1 and #2), and putative *epct*$^{-}$ (#1D, #1E, and #1F) parasites were digested by *Hind* III + *Bam* HI followed by Southern blot analyses using radiolabeled probes for an upstream flanking sequence (5' probe: **A** and **C**) and the open reading frame of *EPCT* (ORF probe: **B** and **D**). The approximate recognition sites of *Hind* III and *Bam* HI and expected DNA fragment sizes are indicated in **E**.

Figure S4. The scheme of Southern blot in Fig. 2. Expected DNA fragment sizes for using the 5' probe (**A**-**B**) or ORF probe of *EPCT* (**C**-**D**) are indicated. TK: thymidine kinase, GFP: green fluorescent protein, SAT: nourseothricin resistance gene.

bioRxiv preprint doi: https://doi.org/10.1101/2023.01.10.523530; this version posted January 11, 2023. The copyright holder for this preprint (which was not certified by peer review) is the author/funder, who has granted bioRxiv a license to display the preprint in perpetuity. It is made available under aCC-BY 4.0 International license.

Figure S5. Chromosomal *EPCT*-null promastigotes cannot lose the pXNG4-EPCT episome. (**A**) *EPCT+/-* + pXNG4-EPCT and *epct*$^{-}$ + pXNG4-EPCT promastigotes were cultivated in the presence of SAT or GCV for 14 passages (as pools) and individual clones were isolated via sorting followed by serial dilution. Plasmid copy number numbers (average ± SDs) were determined by qPCR. (**B**) Promastigotes were cultivated at 27 °C in complete M199 media and culture densities were determined daily using a hemocytometer. Error bars indicate standard deviations from three biological repeats (**: $p < 0.01$).

Figure S6. EPCT overexpression leads to reduced growth in BALB/c mice. Following footpad infection, mice were treated with GCV or PBS and euthanized at the indicated timepoints. (**A**) Mouse body weights were measured at day 0-21 post infection. (**B**) Genomic DNA samples were prepared from lesion-derived amastigotes and parasite loads were determined by qPCR using primers targeting the *L. major* 28S rDNA gene (*: $p < 0.05$, **: $p < 0.01$, ***: $p < 0.001$).

Figure S7. EPCT overexpression leads to attenuated virulence in BALB/c mice. Stationary phase promastigotes were injected into the footpad of BALB/c mice as described in *Materials and Methods*. Footpad lesion sizes were measured using a Vernier caliper (**A**) and parasite loads were determined by qPCR (**B**). **: $p < 0.01$.

Figure S8. EPCT overexpression does not affect the cellular levels of IPC. Total lipids were extracted from stationary phase promastigotes and analyzed by ESI/MS in the negative ion mode using both total ion current scan and precursor ion scan of m/z 241. Error bars represent standard deviations from 4 independent experiments.

Figure S9. EPCT overexpression does not affect mitochondrial ROS production. Log phase promastigotes were cultivated in complete M199 medium **(A, C)** or transferred to PBS **(B, D)**

bioRxiv preprint doi: https://doi.org/10.1101/2023.01.10.523530; this version posted January 11, 2023. The copyright holder for this preprint (which was not certified by peer review) is the author/funder, who has granted bioRxiv a license to display the preprint in perpetuity. It is made available under aCC-BY 4.0 International license.

and labeled with MitoSox Red for 25 min. Mean fluorescence intensity (MFI) for MitoSox Red (**A**, **B**) and percentages of dead cells (**C**, **D**) were determined by flow cytometry at the indicated timepoints. Cell growth rates were determined by hemocytometer counting **(E)**. Error bars represent standard deviations from three independent experiments.

bioRxiv preprint doi: https://doi.org/10.1101/2023.01.10.523530; this version posted January 11, 2023. The copyright holder for this preprint (which was not certified by peer review) is the author/funder, who has granted bioRxiv a license to display the preprint in perpetuity. It is made available under aCC-BY 4.0 International license.

Table S1. List of oligonucleotides used in this study. Sequences in lowercase represent restriction enzyme sites.

Primer	Name	Sequence
#52	5’ UTR EPCT forward	CATGACgaattcGACCAGCAACGTGAGGGAC
#12	5’ UTR EPCT reverse	TCAGACACTAGTGATCATGGATCCGGTGGCGGCAGAAGTGGAAG
#13	3’ UTR EPCT forward	TCAGTAggatccCGTAGTGGGCTGGCGGGGAG
#14	3’ UTR EPCT reverse	GATCATaagcttCAAATAAAGAGAGTCAGTG
#9	EPCT ORF forward	GATCAGggatccACCATGCCCACCGTTTCTTCG
#10	EPCT ORF reverse	GACTACggatccCTAGCTTGCCTCCCGTAATTTG
#73	EPCT 5’ UTR Southern forward	AGCACTTGCTCCAAGCGAAGAG
#74	EPCT 5’ UTR Southern reverse	GAACAAGAAGCCGTACTTCACAG
#15	EPCT ORF Southern forward	GCCGAGGAGCGCTATGAGGC
#16	EPCT ORF Southern reverse	GAGAACTTGTCGCCTACAAC
#699	28S rRNA qPCR forward	AAGATGGACCGGCCTCTAGT
#700	28S rRNA qPCR reverse	ATCCTTCCCCGCTCCAGTAT
#703	pXNG4 qPCR forward	CCCGACAACCACTACCTGAG
#704	pXNG4 qPCR reverse	GTCCATGCCGAGAGTGATCC
#842	EPCT ORF RT forward	CATGAGCTTCAACGAGCGTG
#843	EPCT ORF RT reverse	GCCGTCAATCACATCCTTGC

Supplemental figures

Fig. S1

```
Sc    ------------------------------------------------------------    0
Pf    MSNQFLVDTIYNHGYMRKFLSILRSVKKNDKFQYIMKLCENTNIEEDEELYKVFINELHN    60
Lm    ---------------------------------------------------------M--    1
At    -----------------------------------------------------MVWEK--    5
Hs    ------------------------------------------------------------    0

Sc    ------------------------------------------------------------    0
Pf    ISNTSSTRRKKNDSSCNESTNNTGNNKENGSNNTHFEDMKDINSSISNNSEEINEFEIDS    120
Lm    ---------PTVSSSTPSP----ASTPTVGVPPGKVW----LNADEPND----EYSLFCT    40
At    ---------EKIVGSCIVG----GAAFAVGASFLHLF----LKGELPLG----LGLGLSC    44
Hs    --------------------------------------------MIRNG----RGAAGGA    12

Sc    ---MTVNLDPDKVWIDGCFDFTHHGHAGAILQARRTVSKENGKLFCGVHTDEDIQHNKG-    56
Pf    STSTQEKTKETRIYVDGIFDLSHSGHFNAMRQAKKL----GDIVVVGINSDEDALNSKGV    176
Lm    EAIPPKVPGTVRIWVDGCFDMLHFGHANALRRARRL----GDELFVGCHSDEEVMRFKG-    95
At    PWRILRKRKPVRVYMDGCFDMMHYGHCNALRQARAL----GDQLVV--VSDEEIIANKG-    97
Hs    EQPGPGGRRAVRVWCDGCYDMVHYGHSNQLRQARAM----GDYLIVGVHTDEEIAKHKG-    67
                 ::: ** :*: * ** . : :*:      .. :.    :**:    **

Sc    TPVMNSSERYEHTRSNRWCSEVVEAAPYVTDPN----WMDKYQCQYVVHGDDITIDANGE    112
Pf    KPIYTQEERGALIAGCKWVDEVIIGTKYNVDMD----LLEKYNCDYAAHGTDLAYDKNGT    232
Lm    PPIMHAEERYEALRACKWVDHVVENYPYCTRLK----DIERFEIDYVVHGDDISVDLNGR    151
At    PPVTPLHERMTMVKAVKWVDEVISDAPYAITEDFMKKLFDEYQIDYIIHGDDPCVLPDGT    157
Hs    PPVFTQEERYKMVQAIKWVDEVVPAAPYVTTLE----TLDKYNCDFCVHGNDITLTVDGR    123
       *:    **     . :* ..*:    *    .     ::.:: ::  ** *     :*

Sc    DCYKLVKEMGRFKVVKRTYGVSTTEIIHRILTKKSLPPTHPDYYPTTQE-----------    161
Pf    CCYEEVRKFNKLKIFERSYGISTTTIINHLLQAVNNSN----YYSSSNNNNNNNNNNNNN    288
Lm    NSYQEIIDAGKFKVVKRTKGISTTDLVGRMLLCTKNHM----LKSVDEVQ----------    197
At    DAYALAKKAGRYKQIKRTEGVSSTDIVGRMLLCVRERS----ISDTHSRS----------    203
Hs    DTYEEVKQAGRYRECKRTQGVSTTDLVGRMLLVTKAHH----SSQEMSS-----------    168
        *    . .: :  :*: *:*:* :: ::*                .

Sc    ------------------------------------------L-----------------    162
Pf    NTLVNSNNNNNNNDTNSVSTNEISDINNETNYVYNTNTNSEQLDNFNKNKDNPNILEITE    348
Lm    -------------------------L--E----------------N--------------    200
At    ------------------------SLQRQ----------------F--------------    209
Hs    ----------------------------E----------------Y--------------    170

Sc    -SFYS----VA-------------------QDAVSKHCYVFQRDLDNVLVNGG--YKFDA    196
Pf    EQIYNSELGISDDNKTKVSEQQHDIDTLPKNLLNRNRCHITTSQIYQFIDNNELIKKKKN    408
Lm    -SLL--------------------------EHSPTMPCLTTSRKIVQFSNNSS---PKPG    230
At    -SHGHSSPKFEDGASS--------------AGTRVSHFLPTSRRIVQFSNGKG---PGPD    251
Hs    -REYADSFGKCPGGRN--------------PWTGVSQFLQTSQKIIQFASGKE---PQPG    212
                                                : :.  .

Sc    EDCVYVDGDFDLFHMGDIDQLRKLKMDLHPDKKLIVGITTSD-------YSSTIMTMKER    249
Pf    KKVVYVDGSFDIFHIGHLRILENAKK-LG--DYLLVGMHSDEVVQKMKGKYFPVVSLLER    465
Lm    DRIVYVDGSFDLFHIGHIRVLQKARE-LG--DYVIAGVYEDQVVNEHKGKNYPIMSFNER    287
At    ARIIYIDGAFDLFHAGHVEILRRARE-LG--DFLLVGIHNDQTVSAKRGAHRPIMNLHER    308
Hs    ETVIYVAGAFDLFHIGHVDFLEKVHR-LAERPYIIAGLHFDQEVNHYKGKNYPIMNLHER    271
         :*: * **:** *.:  *.. :  *     ::.*:  .:           ::.: **

Sc    VLSVLSCKYVDAVIIDADATSMSQYNCEKYHIGTAVLTAA--------------------    289
Pf    TLNVLAMKVVDDVVIGAPWVITE-SFIKRFHIDVVVRGTIVDY-IYSNNEIDPYDIPKKL    523
Lm    VLGVLSCRYVDEVVMGVPFDVSK-DVIDGLHINVVVGDKFSDL-VVEEGGSTRYEVPKAM    345
At    SLSVLACRYVDEVIIGAPWEVSR-DTITTFDISLVVHGTVAESDDFRKEEDNPYSVPISM    367
Hs    TLSVLACRYVSEVVIGAPYAVTA-ELLSHFKVDLVCHGKTEIIPD--RDGSDPYQEPKRR    328
       *.**: : *. *::..             .:. .

Sc    GKF-----SEYLTKELIVKRVESQREVYIARNQKKGMSI---------------------    323
Pf    NIYQELSSESNITTYEIIQRIEKNKKY-LMRNMSKRNKKEESIWETSNTYAINN------    576
Lm    GIYHEVDSGCILSTDSLIDRVVENRLDFLKRQAEKRI-KDTKSQEIKPDEYRK-LREAS-    402
At    GIFQVLDSPLDITTSTIIRRIVANHEAYQKRNAKKEA-SEKKYYEQKSFVSGD-------    419
Hs    GIFRQIDSGSNLTTDLIVQRIITNRLEYEARNQKKEA-KELAFLEAARQQAAQPLGERDG    387
      . :        ::.  :: *:  ::     *: .*

Sc    -- 323
Pf    -- 576
Lm    -- 402
At    -- 419
Hs    DF 389
```

Fig. S2

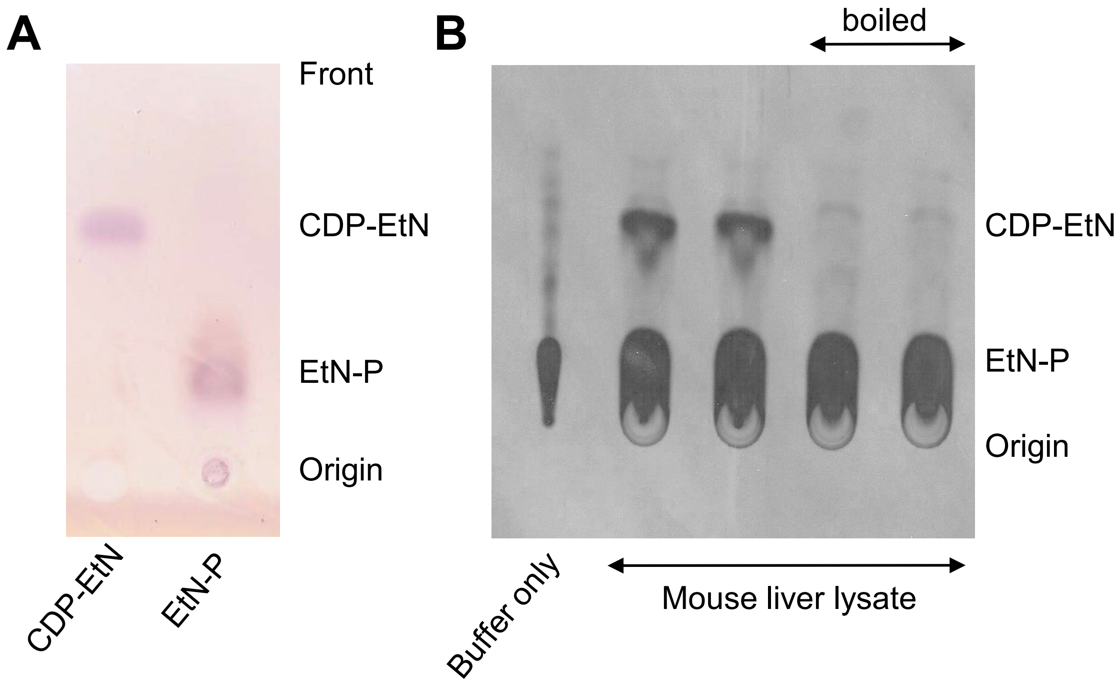
A
Front
CDP-EtN
EtN-P
Origin
CDP-EtN
EtN-P
B
boiled
CDP-EtN
EtN-P
Origin
Buffer only
Mouse liver lysate

Fig. S3

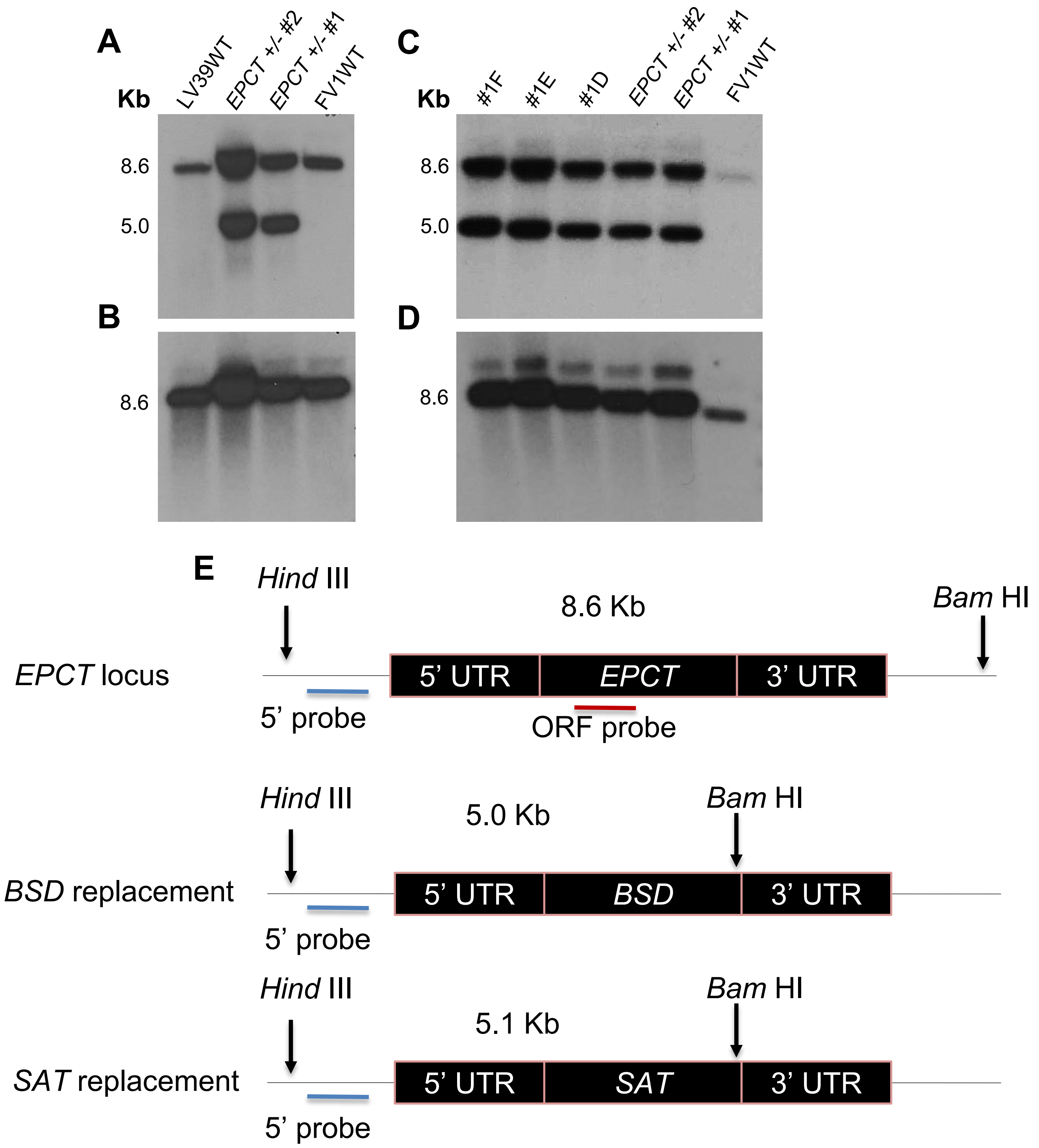

A
Kb
LV39WT
EPCT +/- #2
EPCT +/- #1
FV1WT
8.6
5.0
B
8.6
C
Kb
#1F
#1E
#1D
EPCT +/- #2
EPCT +/- #1
FV1WT
8.6
5.0
D
8.6
E
Hind III
8.6 Kb
Bam HI
EPCT locus
5' UTR
EPCT
3' UTR
5' probe
ORF probe
Hind III
5.0 Kb
Bam HI
BSD replacement
5' UTR
BSD
3' UTR
5' probe
Hind III
Bam HI
5.1 Kb
SAT replacement
5' UTR
SAT
3' UTR
5' probe

Fig. S4

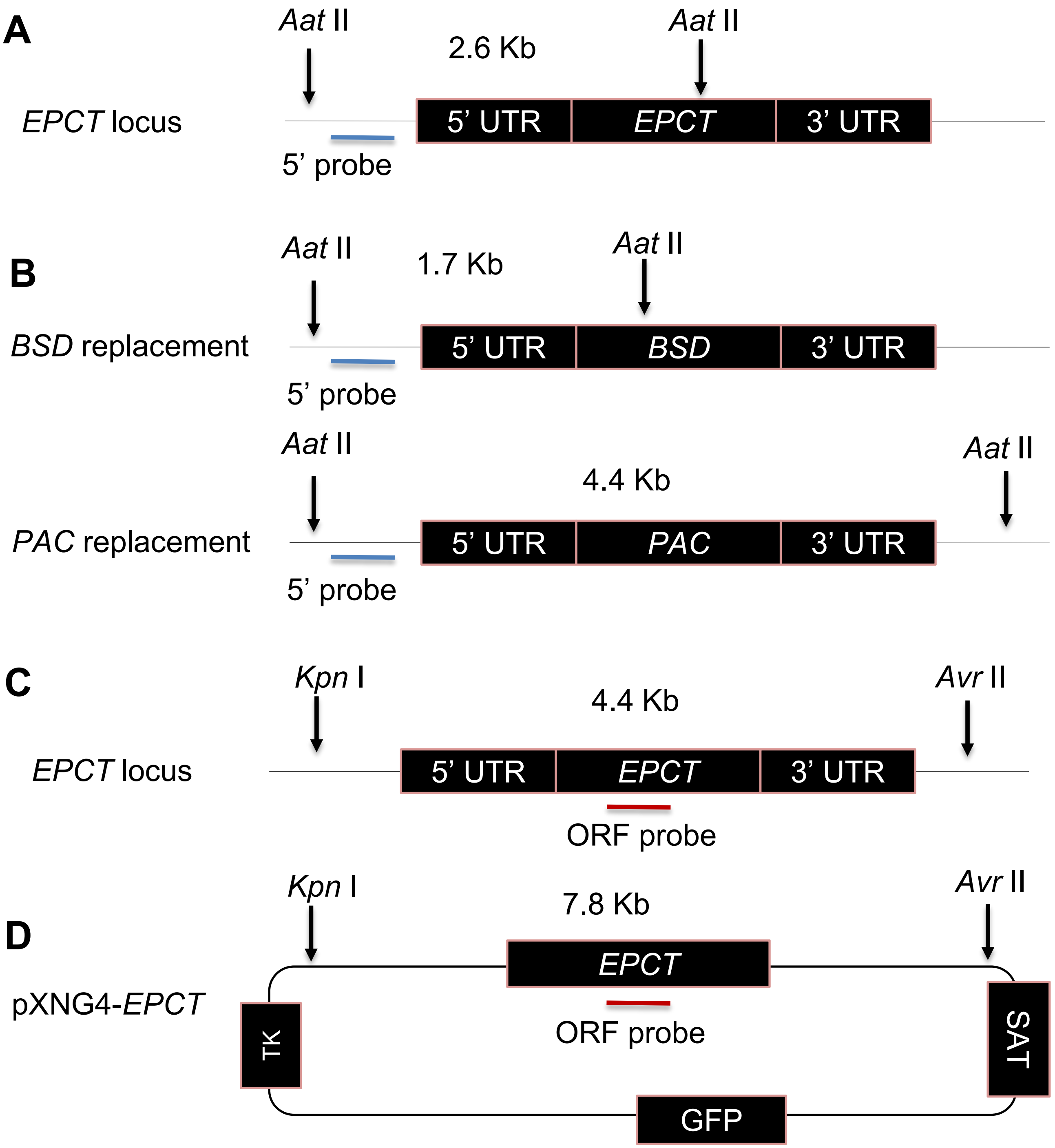
A
Aat II
2.6 Kb
Aat II
EPCT locus
5' UTR
EPCT
3' UTR
5' probe
B
Aat II
1.7 Kb
Aat II
BSD replacement
5' UTR
BSD
3' UTR
5' probe
Aat II
4.4 Kb
Aat II
PAC replacement
5' UTR
PAC
3' UTR
5' probe
C
Kpn I
4.4 Kb
Avr II
EPCT locus
5' UTR
EPCT
3' UTR
ORF probe
D
Kpn I
7.8 Kb
Avr II
pXNG4-EPCT
EPCT
ORF probe
TK
SAT
GFP

Fig. S5

A

Cell Type	*epct*$^{-}$ + pXNG4-*EPCT* + SAT (pool)	*epct*$^{-}$ + pXNG4-*EPCT* + GCV (GFP low clone)	*EPCT*+/- + pXNG4-*EPCT* GCV (GFP low clone)
pXNG4-*EPCT* copies/cell	25 ± 2.1	8.2 ± 1.2	0.0031 ± 0.0020

B

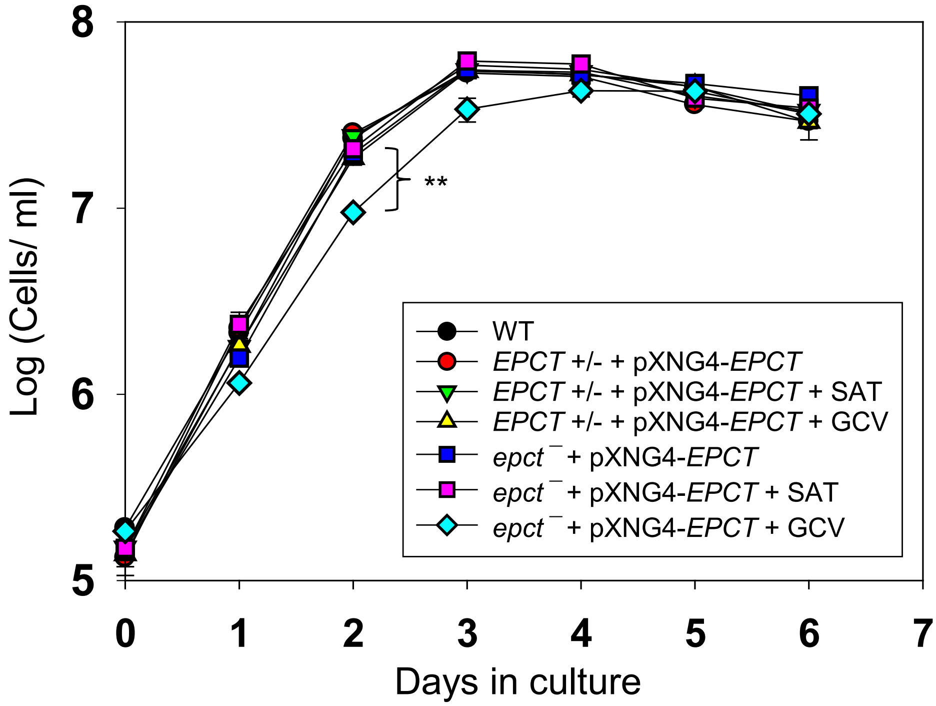

Fig. S6

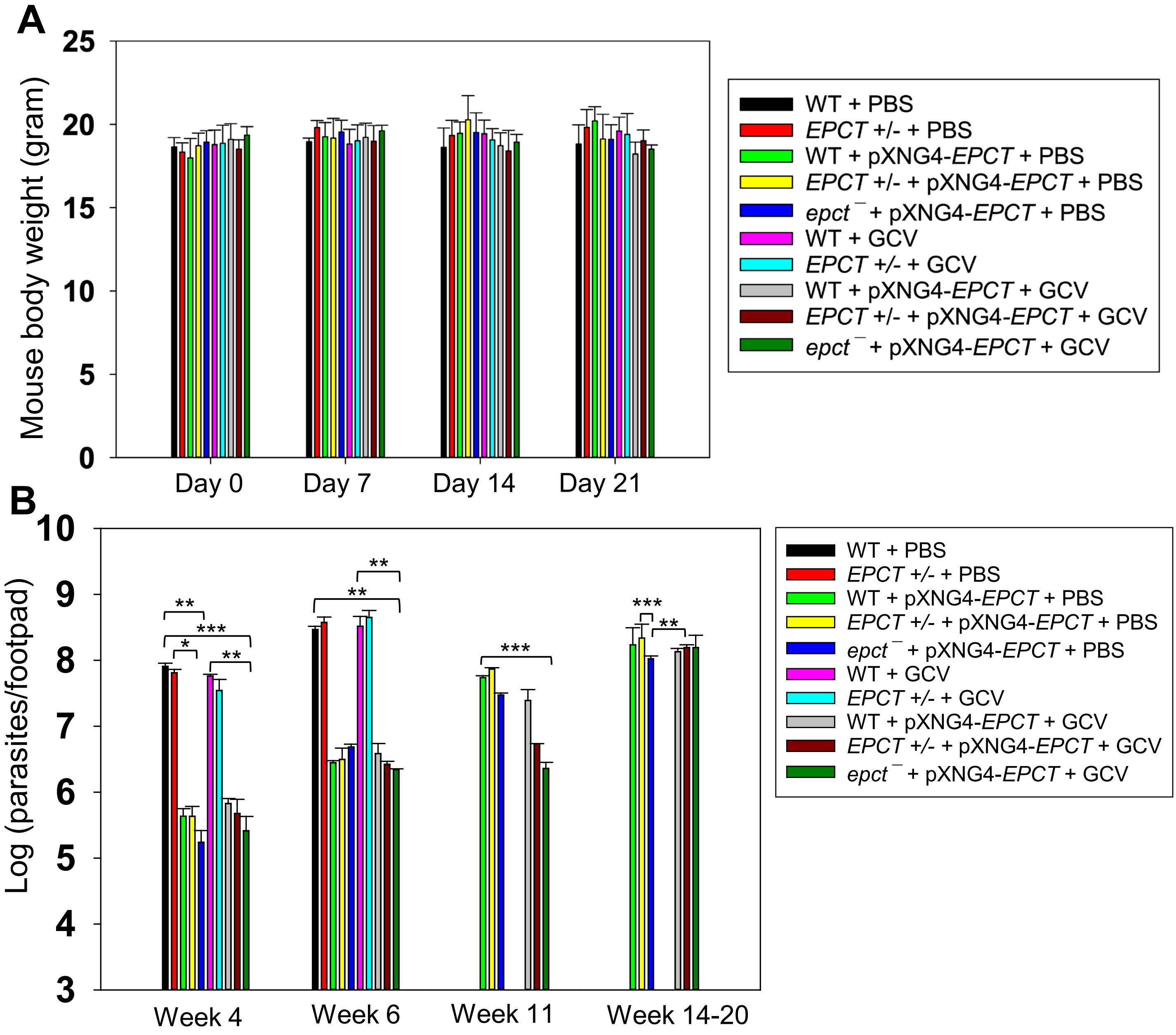
A
Mouse body weight (gram)
0
5
10
15
20
25
Day 0
Day 7
Day 14
Day 21
WT + PBS
EPCT +/- + PBS
WT + pXNG4-EPCT + PBS
EPCT +/- + pXNG4-EPCT + PBS
epct⁻ + pXNG4-EPCT + PBS
WT + GCV
EPCT +/- + GCV
WT + pXNG4-EPCT + GCV
EPCT +/- + pXNG4-EPCT + GCV
epct⁻ + pXNG4-EPCT + GCV
B
Log (parasites/footpad)
3
4
5
6
7
8
9
10
Week 4
Week 6
Week 11
Week 14-20
**
***
*
**
**
**
***
***
**
WT + PBS
EPCT +/- + PBS
WT + pXNG4-EPCT + PBS
EPCT +/- + pXNG4-EPCT + PBS
epct⁻ + pXNG4-EPCT + PBS
WT + GCV
EPCT +/- + GCV
WT + pXNG4-EPCT + GCV
EPCT +/- + pXNG4-EPCT + GCV
epct⁻ + pXNG4-EPCT + GCV

Fig. S7

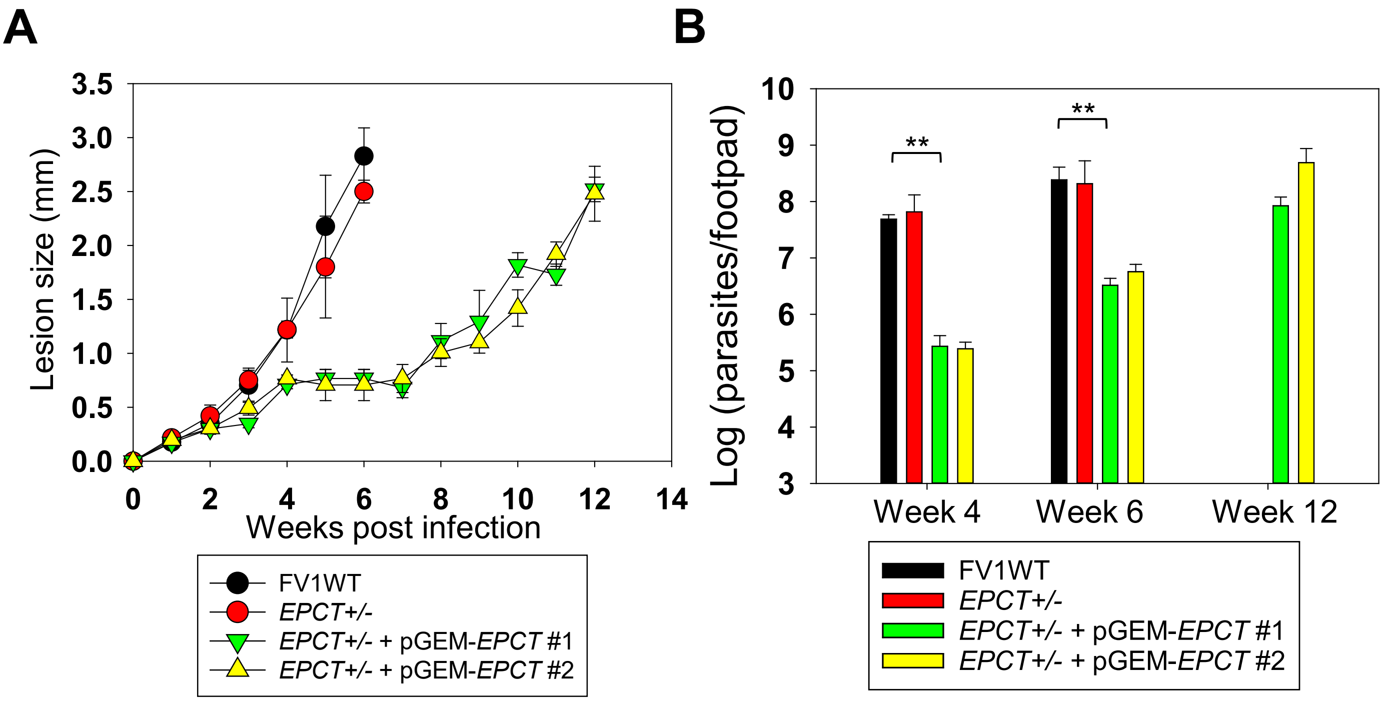
A
3.5
3.0
2.5
2.0
1.5
1.0
0.5
0.0
Lesion size (mm)
0
2
4
6
8
10
12
14
Weeks post infection
FV1WT
EPCT+/-
EPCT+/- + pGEM-EPCT #1
EPCT+/- + pGEM-EPCT #2
B
10
9
8
7
6
5
4
3
Log (parasites/footpad)
**
**
Week 4
Week 6
Week 12
FV1WT
EPCT+/-
EPCT+/- + pGEM-EPCT #1
EPCT+/- + pGEM-EPCT #2

Fig. S8

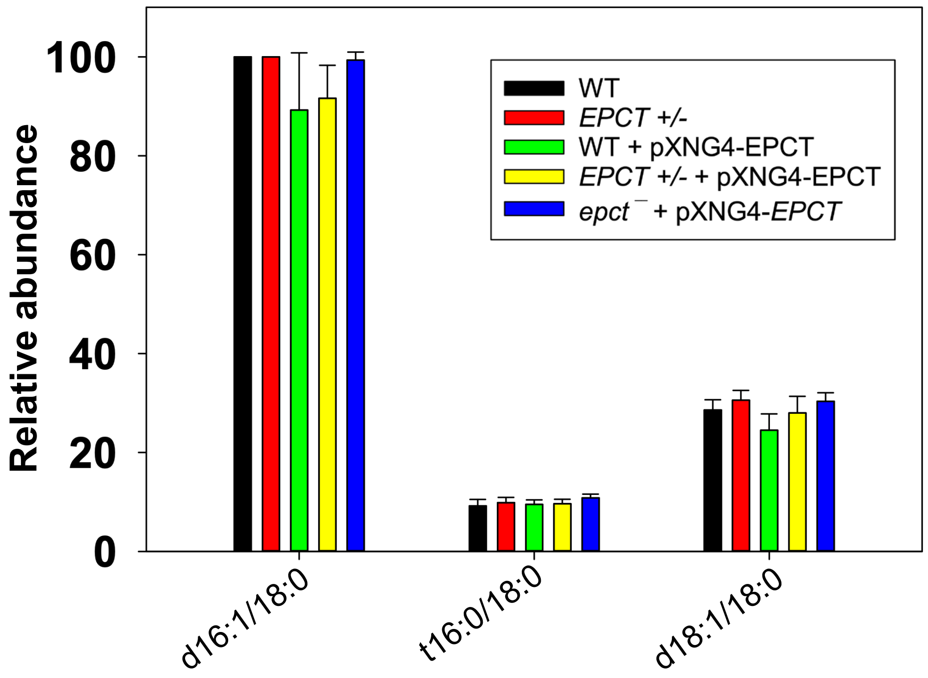
100
80
60
40
20
0
Relative abundance
WT
EPCT +/-
WT + pXNG4-EPCT
EPCT +/- + pXNG4-EPCT
epct⁻ + pXNG4-EPCT
d16:1/18:0
t16:0/18:0
d18:1/18:0

Fig. S9

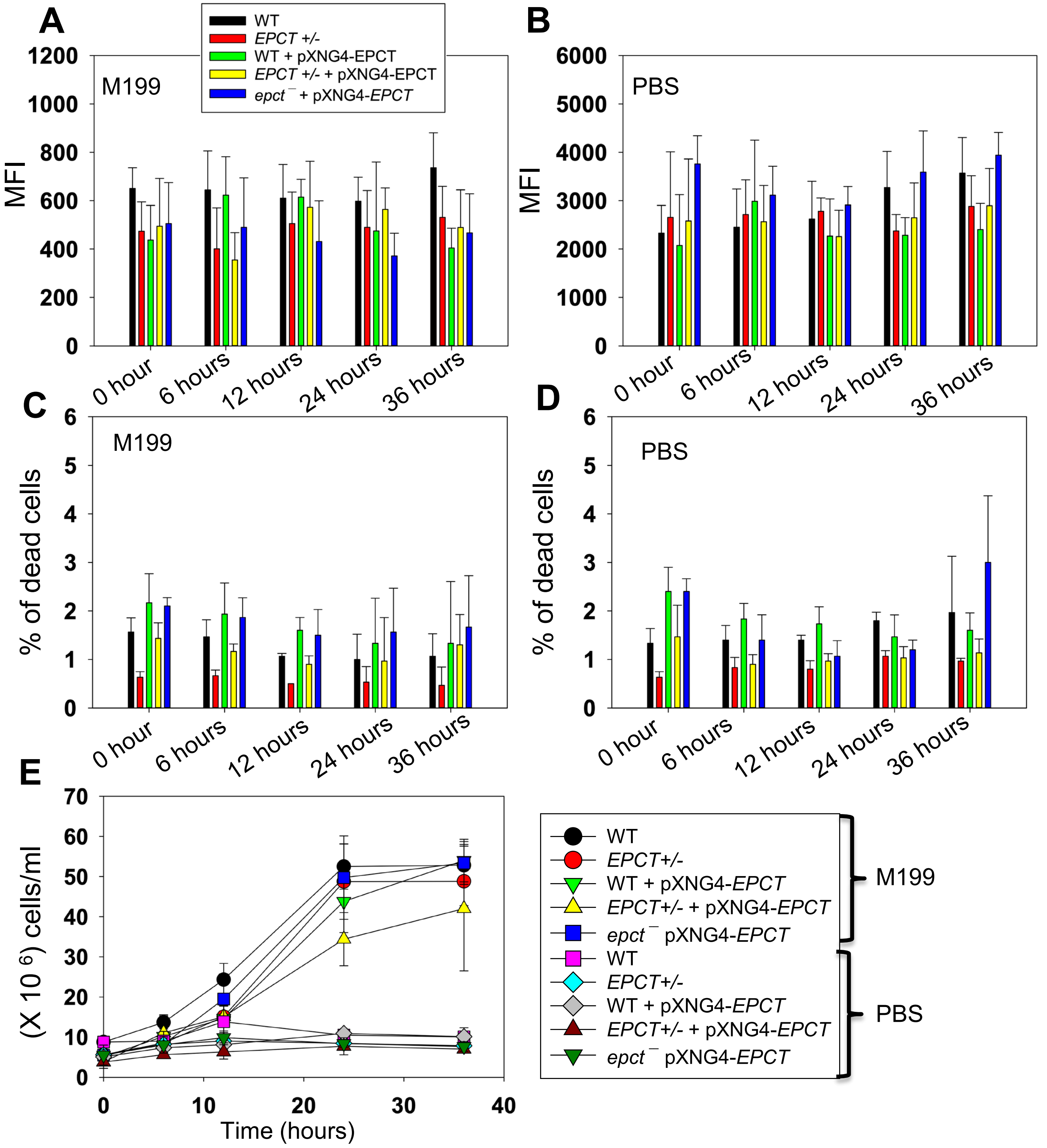

A
B
C
D
E
WT
EPCT +/-
WT + pXNG4-EPCT
EPCT +/- + pXNG4-EPCT
epct⁻ + pXNG4-EPCT
M199
PBS
MFI
% of dead cells
0 hour
6 hours
12 hours
24 hours
36 hours
(X 10 6) cells/ml
Time (hours)
WT
EPCT+/-
WT + pXNG4-EPCT
EPCT+/- + pXNG4-EPCT
epct⁻ pXNG4-EPCT
WT
EPCT+/-
WT + pXNG4-EPCT
EPCT+/- + pXNG4-EPCT
epct⁻ pXNG4-EPCT
M199
PBS
